# Vascular endothelial growth factor receptor 2 as a potential host target for the inhibition of enterovirus replication

**DOI:** 10.1128/jvi.01129-24

**Published:** 2024-09-17

**Authors:** Xiaoyu Zhao, Rui Qiao, Meng Hao, Longfa Xu, Dong Wang, Yinying Lu, Jiayan Li, Jing Wu, Yi Li, Tong Cheng, Wenhong Zhang, Jincun Zhao, Pengfei Wang

**Affiliations:** 1 Shanghai Sci-Tech Inno Center for Infection & Immunity, National Medical Center for Infectious Diseases, Huashan Hospital, Institute of Infection and Health, Fudan University, Shanghai, China; 2 Shanghai Pudong Hospital, Fudan University Pudong Medical Center, State Key Laboratory of Genetic Engineering, MOE Engineering Research Center of Gene Technology, School of Life Sciences, Shanghai Institute of Infectious Disease and Biosecurity, Fudan University, Shanghai, China; 3 Greater Bay Area Institute of Precision Medicine (Guangzhou), Fudan University, Nansha District, Guangzhou, China; 4 State Key Laboratory of Vaccines for Infectious Diseases, Xiang An Biomedicine Laboratory, School of Life Sciences, School of Public Health, Xiamen University, Xiamen, China; 5 State Key Laboratory of Respiratory Disease, National Clinical Research Center for Respiratory Disease, Guangzhou Institute of Respiratory Health, the First Affiliated Hospital of Guangzhou Medical University, Guangzhou, China; 6 Department of Infectious Diseases, Shanghai Key Laboratory of Infectious Diseases and Biosafety Emergency Response, National Medical Center for Infectious Diseases, Huashan Hospital, Shanghai Medical College, Fudan University, Shanghai, China; 7 Human Phenome Institute, Fudan University, Shanghai, China; 8 Guangzhou Laboratory, Bio-Island, Guangzhou, China; 9 Shanghai Institute for Advanced Immunochemical Studies, School of Life Science and Technology, ShanghaiTech University, Shanghai, China; 10 Institute for Hepatology, National Clinical Research Center for Infectious Disease, Shenzhen Third People’s Hospital; The Second Affiliated Hospital, School of Medicine, Southern University of Science and Technology, Shenzhen, China; Loyola University Chicago - Health Sciences Campus, Maywood, Illinois, USA

**Keywords:** EV-A71, kinase inhibitors, VEGFR family, VEGFR2, host factor

## Abstract

Because host kinases are key regulators of multiple signaling pathways in response to viral infections, we previously screened a kinase inhibitor library using rhabdomyosarcoma cells and human intestinal organoids in parallel to identify potent inhibitors against EV-A71 infection. We found that Rho-associated coiled-coil-containing protein kinase (Rock) inhibitor efficiently suppressed the EV-A71 replication and further revealed Rock1 as a novel EV-A71 host factor. In this study, subsequent analysis found that a variety of vascular endothelial growth factor receptor (VEGFR) inhibitors also had potent antiviral effects. Among the hits, Pazopanib, with a selectivity index as high as 254, which was even higher than that of Pirodavir, a potent broad-spectrum picornavirus inhibitor targeting viral capsid protein VP1, was selected for further analysis. We demonstrated that Pazopanib not only efficiently suppressed the replication of EV-A71 in a dose-dependent manner, but also exhibited broad-spectrum anti-enterovirus activity. Mechanistically, Pazopanib probably induces alterations in host cells, thereby impeding viral genome replication and transcription. Notably, VEGFR2 knockdown and overexpression suppressed and facilitated EV-A71 replication, respectively, indicating that VEGFR2 is a novel host dependency factor for EV-A71 replication. Transcriptome analysis further proved that VEGFR2 potentially plays a crucial role in combating EV-A71 infection through the TSAd-Src-PI3K-Akt pathway. These findings expand the range of potential antiviral candidates of anti-enterovirus therapeutics and suggest that VEGFR2 may be a key host factor involved in EV-A71 replication, making it a potential target for the development of anti-enterovirus therapeutics.

**IMPORTANCE:**

As the first clinical case was identified in the United States, EV-A71, a significant neurotropic enterovirus, has been a common cause of hand, foot, and mouth disease (HFMD) in infants and young children. Developing an effective antiviral agent for EV-A71 and other human enteroviruses is crucial, as these viral pathogens consistently cause outbreaks in humans. In this study, we demonstrated that multiple inhibitors against VEGFRs effectively reduced EV-A71 replication, with Pazopanib emerging as the top candidate. Furthermore, Pazopanib also attenuated the replication of other enteroviruses, including CVA10, CVB1, EV-D70, and HRV-A, displaying broad-spectrum anti-enterovirus activity. Given that Pazopanib targets various VEGFRs, we narrowed the focus to VEGFR2 using knockdown and overexpression experiments. Transcriptomic analysis suggests that Pazopanib's potential downstream targets involve the TSAd-Src-PI3K-Akt pathway. Our work may contribute to identifying targets for antiviral inhibitors and advancing treatments for human enterovirus infections.

## INTRODUCTION

Human enterovirus A71 (EV-A71), belonging to the Enterovirus genus of the *Picornaviridae* family, is a significant causative agent of hand, foot, and mouth disease (HFMD), primarily affecting children under 5 years old (1, 2). HFMD is typically a self-limiting disease, whereas some patients may develop various central nervous system complications, even leading to fatal consequences in severe cases (3, 4). In recent years, HFMD outbreaks, particularly in the Asia-Pacific region, including Malaysia, Vietnam, China, and India, have resulted in large numbers of child deaths, making EV-A71 a significant local public health concern (5). Although three monovalent inactivated vaccines against EV-A71 have been approved in China since 2016, the epidemic trend remains beyond full control due to the lack of mucosal immunity, as well as the frequent mutations and recombination of RNA viruses (6). Furthermore, there are currently no clinically approved antiviral drugs targeting EV-A71.

Host protein kinases play a crucial role in modulating the activity, localization, and function of downstream effectors through the reversible phosphorylation of their target proteins, and thus impact various cellular functions, such as proliferation, survival, motility, metabolism, angiogenesis, and evasion of anti-tumor immune responses (7, 8). Apart from the multiple functions of protein kinases in cellular processes, many viruses, such as the Ebola and influenza viruses, rely on cellular kinases for their replication (9, 10). A recent study found that Tribbles pseudokinase 3 (TRIB3) can promote EV-A71 infection through dual mechanisms by maintaining the metabolic stability of Scavenger receptor class B member 2 (SCARB2) to enhance the entry and spread of the virus, as well as by promoting the replication of EV-A71 RNA in a SCARB2-independent manner (11). Misshapen NIK-related kinase (MINK) has also been reported to be associated with the translation efficiency of EV-A71 viral protein synthesis through the stimulation of the p38 mitogen-activated protein kinase pathway (12). In addition, we previously conducted simultaneous screening of a protein kinase inhibitor library by using RD cells and 2D human intestinal organoids to identify cellular kinases required for EV-A71 viral growth. We found that GSK269962A, a Rho-associated coiled-coil-containing protein kinase (Rock) inhibitor, exhibits antiviral effects, and depletion of Rock1 significantly reduced EV-A71 replication, indicating that Rock1 is a host-dependent factor involved in EV-A71 replication (13). Therefore, it is clear that EV-A71 utilizes host factors, including host protein kinases, to complete its life cycle.

Vascular endothelial growth factor (VEGF) is the principal angiogenic growth factor that modulates angiogenesis through receptor tyrosine kinase VEGF receptors (VEGFRs) (14). Multiple VEGFs (VEGF-A, VEGF-B, VEGF-C, and VEGF-D) interact with different VEGFRs, such as VEGFR1, VEGFR2, and VEGFR3. These VEGFRs display similar structural features but differ in activation mode, signal transduction, and biological functions (15). For instance, VEGFR1, VEGFR2, and VEGFR3 are essential for the development of hematopoietic cells, vascular endothelial cells and lymphatic endothelial cells, respectively. Structurally, each VEGFR consists of three parts, including seven immunoglobulin-like domains in the extracellular domain, a single receptor transmembrane region, and a consensus tyrosine kinase sequence interrupted by a kinase insert domain (16). When VEGF binds to VEGFR, the tyrosine residues in its intracellular signal transduction region are immediately phosphorylated, activating the intracellular downstream signaling pathways, such as p38/MAPK, RAS/RAF/MEK/ERK, and PI3K/AKT/mTOR. This activation leads to the growth, proliferation, and maturation of vascular endothelial cells, and the formation of new blood vessels. Furthermore, VEGFA/VEGFR2 signaling seems to prominently mediate cellular responses involved in angiogenesis. However, dysfunctional VEGF–VEGFR signaling can lead to many human diseases, especially tumors (17, 18). Currently, inhibitors targeting VEGFRs, have shown therapeutic effects on various types of solid tumors. Taken together, VEGFRs play an important role in the regulation of tumor-induced angiogenesis and are thus a therapeutic target for tumor therapy.

At present, the role of VEGFRs in viral replication is rarely reported. Previous studies have shown that infections, such as herpes simplex virus (HSV-1) and Andes virus (ANDV), utilize cellular signaling pathways to regulate the expression of VEGFR2 (19, 20). For instance, corneal infection with HSV-1 can cause herpetic stromal keratitis (HSK), leading to angiogenesis, visual impairment, and blindness (21). VEGF and VEGFR2 are involved in the pathogenesis of HSK by inducing lymphangiogenesis in the cornea and underlying stromal tissue (19). The hantavirus pulmonary syndrome caused by ANDV infection is also mediated by the VEGFR2-Src-VE-cadherin pathway, whereas inhibiting VEGFR2 and Src family kinases can block ANDV-induced endothelial cell permeability (20). Recently, Wan et al. found that intracellular VEGFR2 is increased in DENV-infected patients, and the addition of brivanib alaninate inhibited dengue virus proliferation through the VEGFR2/AMPK pathway (22). In this study, we observed a significant reduction in EV-A71 replication following the pharmacological inhibition of VEGFRs. Moreover, VEGFR2 expression was induced in EV-A71-infected cells. These findings prompted us to conduct a series of *in vitro* studies to investigate the role of VEGFR2 in the replication of human enterovirus. Our study identified VEGFR2 as a novel host factor for human enterovirus replication, suggesting it as a potential therapeutic target against human enterovirus infections.

## MATERIALS AND METHODS

### Cell lines, viruses and drugs

RD cells, 293T cells, Vero-E6 cells, Huh7 cells, HepG2 cells, HT-29 cells, Caco-2, BGMK cells, and MRC-5 cells were maintained in Dulbecco’s modified Eagle’s medium (DMEM) (Gibco) and supplemented with 10% fetal bovine serum (FBS) (Gibco) and 100 units/mL penicillin and streptomycin (P/S) at 37°C with 5% CO_2_. THP-1 cells were maintained in Roswell Park Memorial Institute (RPMI) 1640 (Gibco) supplemented with 10% FBS, and 100 units/mL P/S at 37°C with 5% CO_2_. HUVEC cells were maintained in endothelial cell medium (ECM) supplemented with 5% FBS, 1% endothelial cell growth supplement and 100 units/mL P/S at 37°C with 5% CO_2_.

Clinical isolates of enterovirus A71 (EV-A71), coxsackievirus A10 (CVA10), coxsackievirus B1 (CVB1), enterovirus D70 (EV-D70), and human rhinovirus A (HRV-A) were generously provided by Dr. Jingcun Zhao. EV‐A71, CVA10, and EV-D70, were propagated in RD cells, whereas CVB1 and HRV-A were propagated in BGMK cells and MRC-5 cells, respectively. The titers of all virus stocks were determined by 50% tissue culture infectious dose (TCID_50)_ assay as described elsewhere (23). All experiments with live viruses were conducted in biosafety level 2 laboratories upon institutional approval.

Pirodavir (CAS No. 124436–59-5), Pazopanib (CAS No. 444731–52-6), Brivanib (CAS No. 649735–46-6), SU14813 (CAS No. 627908–92-3), Axitinib (CAS No. 319460–85-0), and Dasatinib (CAS No. 302962–49-8) were all purchased from MedChemExpress. MK-2206 dihydrochloride (CAS No. A3010) was purchased from Apexbio.

### RNA extraction and RT-qPCR analysis

Detection of cellular mRNA expression and viral load was performed as described previously (24). In brief, the infected or mock-infected cells were lysed and applied to total RNA extraction by using the MiniBEST Universal RNA Extraction Kit (TaKaRa, Code No.9767). Viral RNA in the supernatant was extracted with the MiniBEST Viral RNA Extraction Kit (TaKaRa, Code No.9766). Reverse transcription was performed using the PrimeScript RT reagent Kit (TaKaRa, Code No.PR037A) with an oligo (dT) primer. The resultant cDNAs were used for qPCR assay with the Light Cycler 480 SYBR Green I Master Mix (Roche) to measure the mRNA expression level of cellular genes or viral load in supernatants. The relative viral gene copy was determined by RT-qPCR, using GAPDH as an internal reference. The primer sequences used in qPCR assay were shown in Table S1.

### Virus titration

The viral titer in the indicated samples was determined by TCID_50_ assay as described previously (25). Briefly, serially diluted samples from 10^−1^ to 10^−8^ in DMEM were inoculated to RD cells, BGMK cells, or MRC-5 in 96‐well plates. The cells were then incubated for 2 to 3 days at 37°C. TCID_50_ was calculated by counting the wells with cytopathic effect in infected cells using the Reed–Muench method.

### Western blot analysis

Western blot analysis was performed as described previously (26). In brief, the indicated cell samples were lysed in RIPA buffer supplemented with protease inhibitors (Thermo Fisher Scientific, 78430). The cell lysates were then separated in 10% SDS-PAGE and transferred to a 0.45-µm nitrocellulose membrane. After blocking, the membrane was incubated with an against VP1 antibody (GeneTex, GTX132339), VEGFR1 antibody (Abcam, ab32152), VEGFR2 antibody (Abcam, ab134191), phosphorylated VEGFR2 antibody (Tyr951) (CST 4991), VEGFR3 antibody (Beyotime, AF6918), Akt antibody (CST 4685), phosphorylated Akt antibody (CST 4060), or GAPDH antibody (GeneTex, GTX627408) diluted 1:5,000 in primary antibody dilution buffer overnight at 4°C, followed by corresponding secondary staining. The blots were visualized by western horseradish peroxidase substrate and imaged with Gel Doc XR+.

### Immunofluorescence staining

Immunostaining analysis was performed according to the standard protocol as described previously (27). In brief, RD cells seeded on an eight-well glass coverslip were inoculated with indicated MOI of EV‐A71. At 24 h post-infection (hpi), the infected or mock‐infected cells were fixed with 4% paraformaldehyde for 30 min. Followed by an additional washing with PBS, the RD cells were permeabilized with 0.2% Triton X-100 and blocked with 0.5% BSA in phosphate-buffered saline (PBS) for 1 h. The cells were then incubated with rabbit anti‐VP1 antibody (GeneTex, GTX132339) overnight at 4°C, followed with FITC‐conjugated goat anti‐rabbit IgG (Beyotime, P0176). Nuclei were counterstained with 4′‐6‐diamino‐2‐phenylindole (DAPI) (GeneTex, GTX132339). The slides were mounted with antifade reagent and imaged with a Carl Zeiss LSM 880 confocal microscope.

### Flow cytometry

Flow cytometry analysis was performed according to the standard protocol as described previously (28). In brief, the infected or mock-infected RD cells were dissociated with 10 mM EDTA and then fixed with 4% paraformaldehyde for 30 min. After permeabilization, the cells were incubated with rabbit anti‐VP1 antibody (GeneTex, GTX132339), followed by labeling with FITC-conjugated goat anti-rabbit IgG (Beyotime, P0176). Flow cytometry analysis was performed using a BD Fortessa flow cytometer (BD Biosciences), and the data were analyzed using FlowJo v.10 (Tree Star, USA).

### Time-of-drug addition assay

RD cells were seeded into 24-well plates or eight-well chamber slides (Millicell) 1 day before infection with EV-A71 at an MOI of 1. After adsorption for 2 h, the cells were washed with PBS once to remove the unbound virus and maintained in DMEM supplemented with 2% FBS. Pazopanib (10 µM) was added to the media at indicated time points (−2 h, 0 h, 2 h, 4 h, 6 h, 8 h, or 10 h) after inoculation. For the time point of −2 h, the RD cells were pretreated with Pazopanib for 2 h before infection. For the time point of 0 h, Pazopanib and EV-A71 were added together and kept in the fresh media afterwards. At 12 hpi, the virus from the culture supernatant were collected, and the relative viral gene copy was detected by RT-qPCR. Meanwhile, the virus titer was assessed using TCID_50_ assay as described before. To analyze VP1 expression, the cells in the eight-well chamber slides were fixed and then monitored using immunofluorescence staining.

### Virus attachment/ entry assay

For the attachment assay, the RD cells were seeded into a 24-well plate and pre-treated with Pazopanib (10 µM) or not for 4 h at 37°C, followed by thorough washing and transfer to a 4°C environment for incubation with EV-A71 at an MOI of 5. After 2 hpi, the infectious inoculum was removed, and cell lysates were collected. The intracellular viral RNA load was then determined using RT-qPCR analysis. For the entry assay, the RD cells were infected with a mixture of EV-A71 at an MOI of 5 and Pazopanib (10 µM) or not for 2 h, followed by thorough washing. The cell lysates were then collected to detect the intracellular viral gene copy by RT-qPCR analysis.

### Small interfering RNA (siRNA) knockdown

Silencer select siRNA targeting human vascular endothelial growth factor receptor 1 (VEGFR1), vascular endothelial growth factor receptor 3 (VEGFR3), and scrambled siRNA were obtained from Thermo Fisher Scientific. ON‐TARGETplus siRNA of human vascular endothelial growth factor receptor 2 (VEGFR2) was obtained from Dharmacon Research. Transfection of indicated siRNA on HUVEC cells was performed using Lipofectamine RNAiMAX Transfection Reagent (Thermo Fisher Scientific, 13778075), following the manufacturer’s manual. In brief, the HUVEC cells were transfected with 50 nM specific siRNA for 2 consecutive days. At day 3 after siRNA transfection, the cells were inoculated with EV‐A71 at an MOI of 0.01 for 1 h at 37°C. At 24 hpi, the cell lysates and supernatant were collected for the following experiment.

### Overexpression experiment

Plasmid expressing human VEGFR2 (pVEGFR2) and corresponding empty vector control (pEmpty vector) were purchased from Sino Biological Inc. (Beijing, China). Plasmid expressing human Akt (pAkt, P42793) was purchased from MiaoLingBio, China. Transfection of indicated plasmids on 293T cells was performed using Lipofectamine 3000 Transfection Reagent (Thermo Fisher Scientific, L3000008), following the manufacturer’s manual. In brief, the 293T cells were transfected with the abovementioned plasmids. After 24 h of transfection, 293T cells were mock-infected or infected with EV-A71 at an MOI of 0.01 for 24 h. The cells and supernatants were then harvested for Western blot analysis and RT-qPCR analysis with specific antibody and primers, respectively.

### Transcriptome analysis

The RNA-Seq data were examined with the quality control of sequencing quality and contaminations by Fastqc. Then, we used the Kallisto software to quantify the abundances of transcripts, which was based on the pseudoalignment for rapidly determining the compatibility of reads with *Homo sapiens* cDNA sequences (29). The transcript-level abundance data from Kallisto were aggregated into gene-level abundance through R package ‘tximport’ (30). The differential expression analysis was conducted through R package ‘DESeq2’ (31). Differentially expressed genes were subject to pathway enrichment analysis Metascape (32).

### Data and statistical analysis

Unpaired t test was performed for data analysis using GraphPad Prism 8.0. A *P* value of <0.05 was considered statistically significant. Data are presented as the mean and standard deviation (SD) of representative experiments. The principal component analysis and correlation analysis were conducted in Rstudio.

## RESULTS

### Inhibition of EV-A71 infection *in vitro* by VEGFR inhibitors

**Fig 1:**
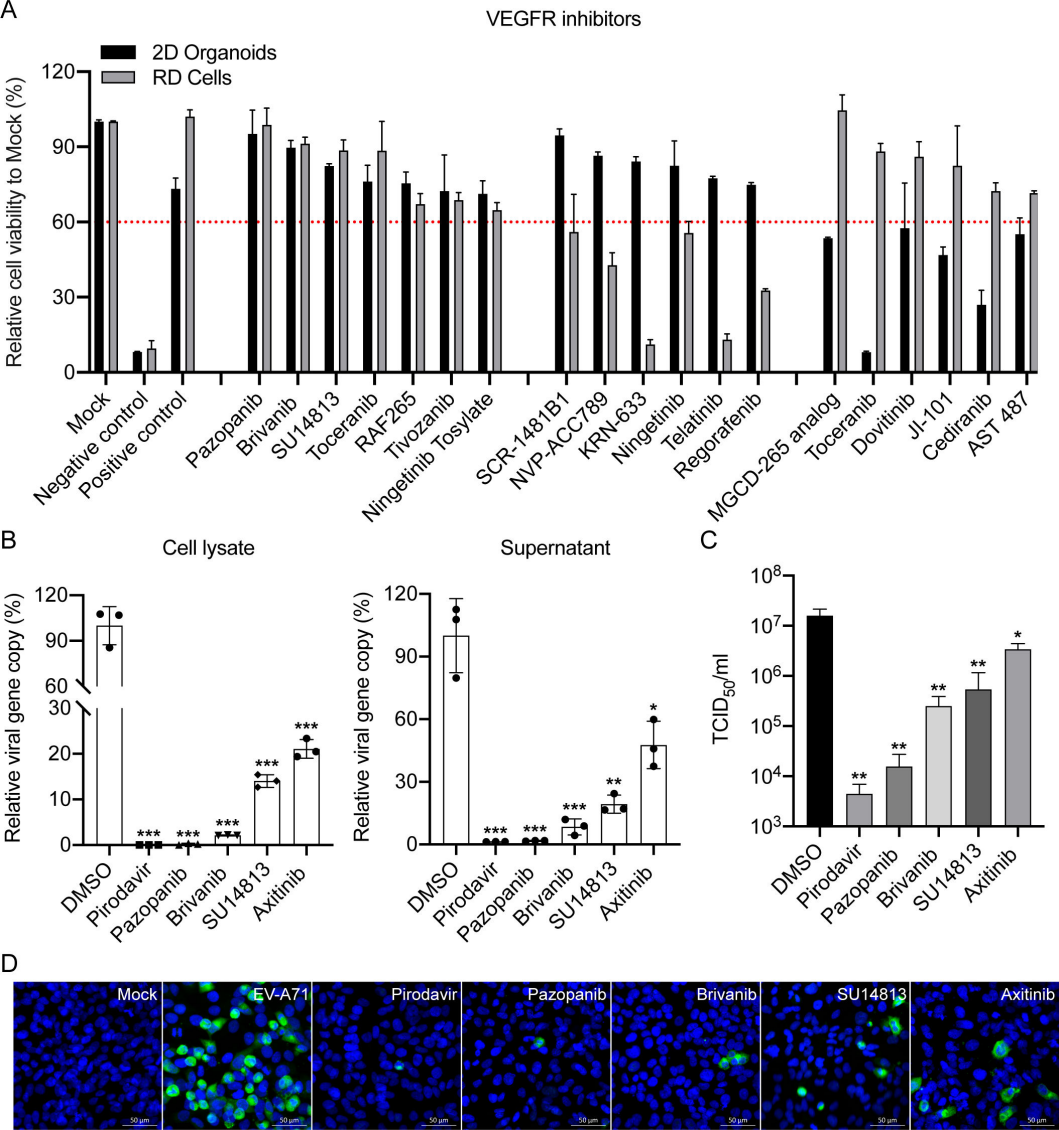
VEGFR inhibitors significantly suppress EV-A71 replication. (**A**) RD cells and 2D human intestinal organoids were inoculated with EV‐A71 at an MOI of 0.01 and 1.25 for 48 h, respectively. The results represent cell viability in the EV-A71-infected RD cells or 2D human intestinal organoids treated with DMSO (Negative control), Pirodavir (Positive control), or indicated VEGFR inhibitor (10 µM) relative to those in mock-infected RD cells or 2D human intestinal organoids. Data show the mean and SD of three independent experiments. (**B**) RD cells treated with the selected inhibitors (10 µM) were inoculated with EV-A71 at an MOI of 0.01 for 48 h. Significant inhibition of EV-A71 infection was found for both cell lysate (left) and supernatant (right) using RT-qPCR analysis. The results represent the viral load in the cells treated with indicated inhibitors relative to that in DMSO-treated cells. (**C**) Viral titer was also determined for inhibitor-treated samples in TCID_50_ assays. Data show the mean and SD of three independent experiments. *P < 0.05; **P < 0.01; ***P < 0.001. (**D**) Representative images of immunofluorescence assay for selected inhibitors using Rabbit anti‐VP1 antibody (green). Nuclei are counterstained with DAPI (blue). Scale bar, 50 µm.

We previously screened a kinase inhibitor library using RD cells and 2D human intestinal organoids in parallel and found that Rock1 is a novel host dependency factor for EV-A71 replication (13). Further analysis found that a variety of VEGFR inhibitors also had potent antiviral effects (Fig. 1A). Among the hits, three representative compounds, Pazopanib, Brivanib, and SU14813, displayed the best cell protection efficiency in both EV‐A71‐infected RD cells, and 2D human intestinal organoids and were selected for antiviral activity experiments. Meanwhile, Pirodavir, a broad‐spectrum anti-picornavirus agent targeting viral capsid protein VP1, and Axitinib, a Food and Drug Administration (FDA)-approved VEGFR inhibitor, were added as positive controls (33).

To investigate whether these VEGFR inhibitors possess antiviral activity against EV-A71, we initially conducted a cytopathic effect (CPE) inhibition assay to evaluate their 50% cytotoxicity concentration (CC_50_) and half maximal effective concentration (EC_50_) in RD cells (Table 1; Fig. S1A). We found that the selectivity index of Pazopanib (254.04) was even higher than that of Pirodavir (120.14), indicating the potential of Pazopanib as a repurposed drug for EV-A71 infection. Next, we performed the viral load reduction assay and found that these VEGFR inhibitors significantly suppressed EV-A71 replication, as evidenced by markedly reduced viral loads in both cell lysate and supernatant (Fig. 1B). Moreover, the viral titers in the supernatant confirmed that these VEGFR inhibitors significantly decreased viral replication (Fig. 1C). In addition, EV‐A71 antigen expression was significantly reduced in VEGFR inhibitor-treated RD cells compared with those treated with DMSO, as evidenced by immunofluorescence staining images and Western blotting (Fig. 1D; Fig. S1B). Overall, these results demonstrated that VEGFR inhibitors efficiently suppressed the replication of EV‐A71, with Pazopanib displaying the most potent antiviral activity among them.

**TABLE 1:** Antiviral activity against EV-A71, cytotoxicity, and selectivity index

Tested compound	CC_50_ (μM)^*a*^	EC_50_ (μM)^*b*^	SI^*c*^
Pirodavir	52.86 ± 1.25	0.44 ± 0.08	120.14
Pazopanib	226.1 ± 27.55	0.89 ± 0.02	254.04
Brivanib	41.29 ± 2.83	1.94 ± 0.33	21.28
SU14813	26.35 ± 6.09	1.73 ± 0.18	15.23
Axitinib	104.0 ± 17.58	4.54 ± 1.17	22.91

^*a*^ CC_50_, compound concentration required to reduce cell viability by 50%.

^*b*^ EC_50_, compound concentration required to achieve 50% protection from virus-induced cytopathogenicity.

^*c*^ SI (selectivity index), ratio CC_50_/EC_50_.

### Antiviral activity of Pazopanib against EV‐A71

**Fig 2:**
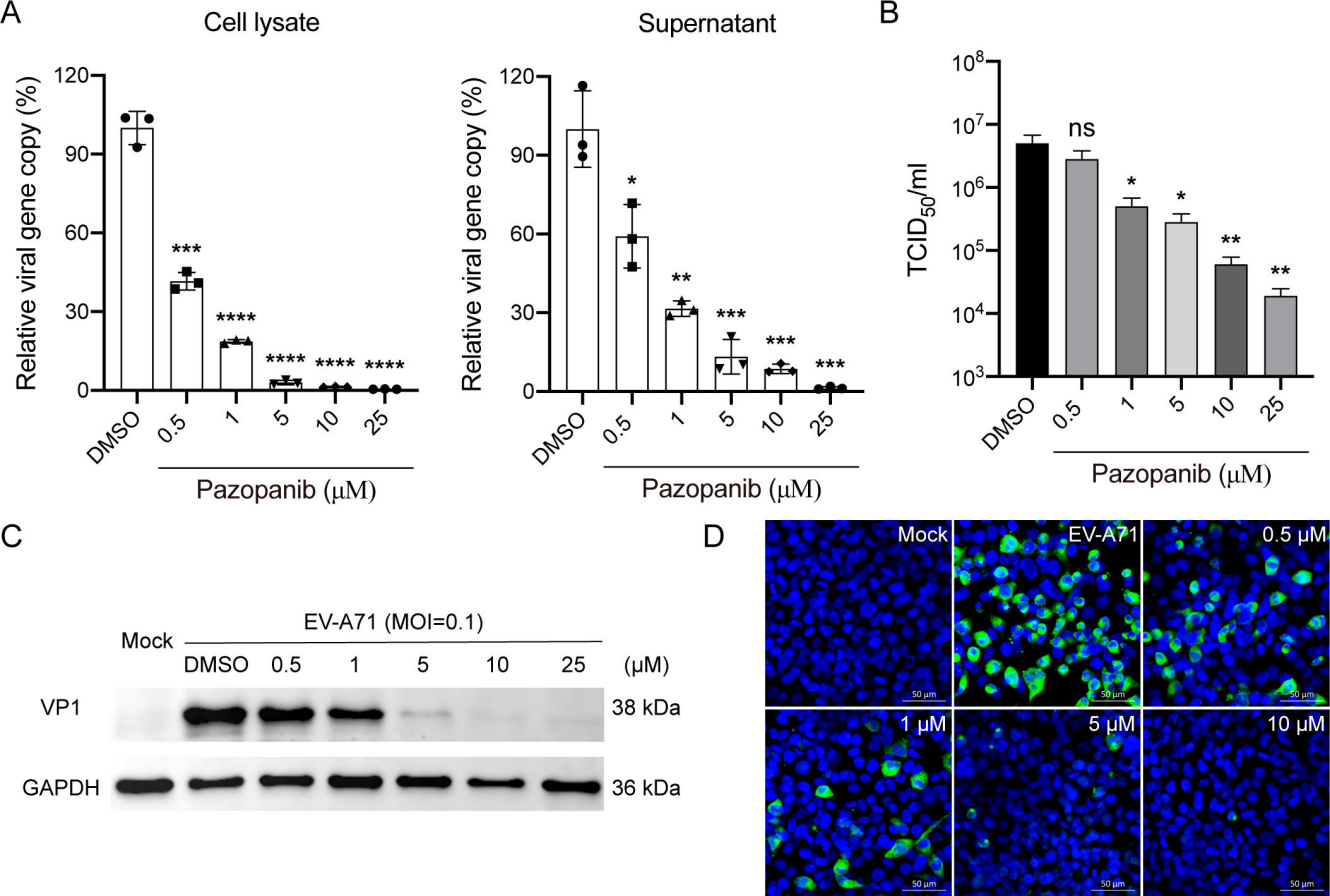
Dose‐dependent inhibition of EV‐A71 by Pazopanib. RD cells treated with the indicated concentration of Pazopanib were inoculated with EV‐A71 at an MOI of 0.1 for 24 h. (**A**) Dose-dependent inhibition of EV-A71 infection was found for both cell lysate (left) and supernatant (right) using RT-qPCR analysis. The results represent the viral load in the cells treated with indicated concentration of Pazopanib relative to that in DMSO-treated cells. (**B**) Viral titer was also determined for samples treated with indicated concentrations of Pazopanib in TCID_50_ assays. Data show the mean and SD of three independent experiments. ns, not statistically significant; *P < 0.05; **P < 0.01; ***P < 0.001. ****P < 0.0001. (**C**) VP1 protein levels were measured for samples treated with indicated concentrations of Pazopanib using Western blotting. (**D**) Representative images of dose-dependent immunofluorescence assay for Pazopanib, using Rabbit anti‐VP1 antibody (green). Nuclei are counterstained with DAPI (blue). Scale bar, 50 µm.

To assess the dose-dependent inhibition of Pazopanib on EV-A71 replication, we evaluated the antiviral activity of Pazopanib in RD cells using various noncytotoxic concentrations. As shown in Fig. 2A and B, the viral load and viral titer of EV‐A71 were markedly reduced in a dose‐dependent manner by Pazopanib in both cell lysates and supernatant, respectively. Furthermore, dose-dependent inhibition of EV-A71 structural protein VP1 expression by Pazopanib was demonstrated in RD cells (Fig. 2C). Similarly, Brivanib, SU14813, and Axitinib also demonstrated dose-dependent inhibition of EV-A71 replication (Fig. S2 and S3). Moreover, immunofluorescence staining further verified that Pazopanib greatly reduced EV-A71 antigen expression in a dose-dependent manner (Fig. 2D). These results revealed that Pazopanib inhibited the replication of EV-A71 in RD cells in a dose-dependent manner.

**Fig 3:**
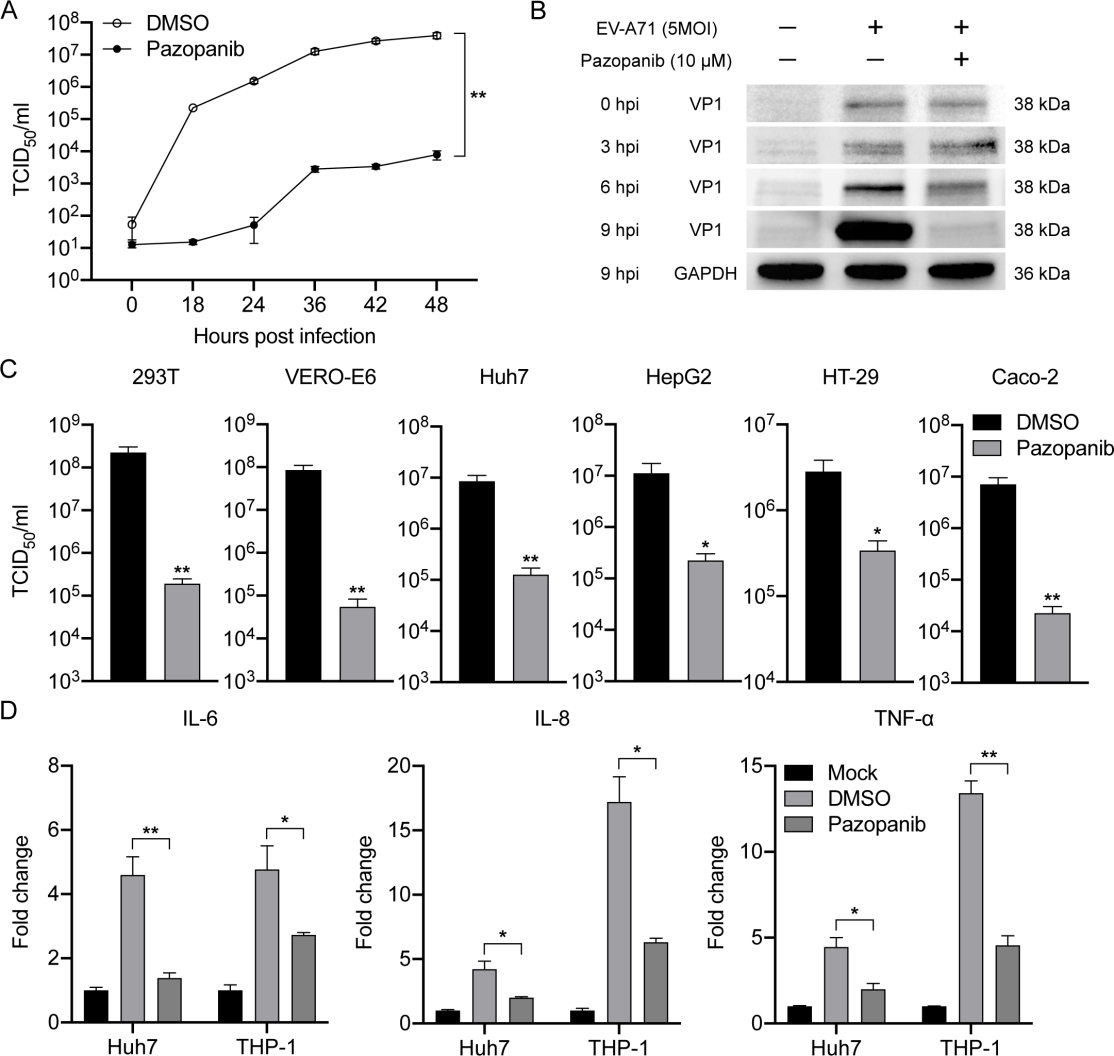
Pazopanib exerts antiviral activity to varying degrees. (**A**) Multi-cycle EV‐A71 growth assay was conducted in the presence or absence of Pazopanib (10  µM). RD cells were infected with EV‐A71 at an MOI of 0.01. Viral titers in cell culture supernatants were quantified by TCID_50_ assay at indicated time points. Differences between the DMSO (open circle) and Pazopanib (black circle) groups were analyzed by Student’s *t*-test. (**B**) Western blot showed reduced EV‐A71 VP1 production after Pazopanib (10 µM) treatment. RD cells were infected with EV‐A71 at an MOI of 1. (**C**) Pazopanib significantly reduced EV‐A71 replication in cell culture supernatants of 293T (MOI = 1), Vero-E6 (MOI = 1), Huh7 (MOI = 1), HepG2 (MOI = 1), HT-29 (MOI = 2), and Caco2 (MOI = 2) at 24 hpi. (**D**) The EV-A71-infected Huh-7 (MOI = 2) or THP-1 (MOI = 5) cells were treated with Pazopanib (10 µM) or mock-treated in triplicate. At 12 hpi, the cells were lysed for detecting mRNA expression levels of proinflammatory cytokines and chemokines. IL-6, interleukin 6; IL-8, interleukin 8; TNF-α, tumor necrosis factor-α. Results show the fold change of GAPDH-normalized expression level in the treated or mock-treated cells relative to that in the mock-infected cells. Data show the mean and SD of three independent experiments. *P < 0.05 **P < 0.01.

Next, the multi-cycle virus growth assay was conducted to assess the virus replication kinetics with or without Pazopanib. As shown in Fig. 3A, viral titers in the cell supernatant decreased dramatically by over 4 logs during the entire time-course with Pazopanib treatment compared with those treated with DMSO. Meanwhile, the expression of the EV-A71 structural protein VP1 was significantly decreased upon addition of Pazopanib, particularly at 9 hpi, as evidenced by Western blotting (Fig. 3B). Flow cytometry analysis also revealed that the percentage of EV-A71-infected RD cells markedly decreased at both 6 and 9 hpi after Pazopanib treatment (Fig. S4A and B). Consistently, the abundance of VP1, as shown by mean fluorescence intensity, was significantly higher in EV-A71-infected RD cells than in those treated with Pazopanib (Fig. S4C). Notably, Pazopanib also significantly reduced EV-A71 replication in multiple cell types, including kidney cells (293T and Vero-E6), liver cells (Huh-7 and HepG2), and colon cells (HT-29 and Caco-2) (Fig. 3C). Additionally, Pazopanib significantly suppressed EV-A71-induced pro-inflammatory cytokine activation in both Huh7 cells and THP1 cells (Fig. 3D). Collectively, these results demonstrated that Pazopanib exhibited potent anti-EV-A71 activity in cell cultures with significant inhibition of virus replication, cell protection, and anti-inflammatory responses.

### Pazopanib exhibits broad-spectrum antiviral effects

**Fig 4:**
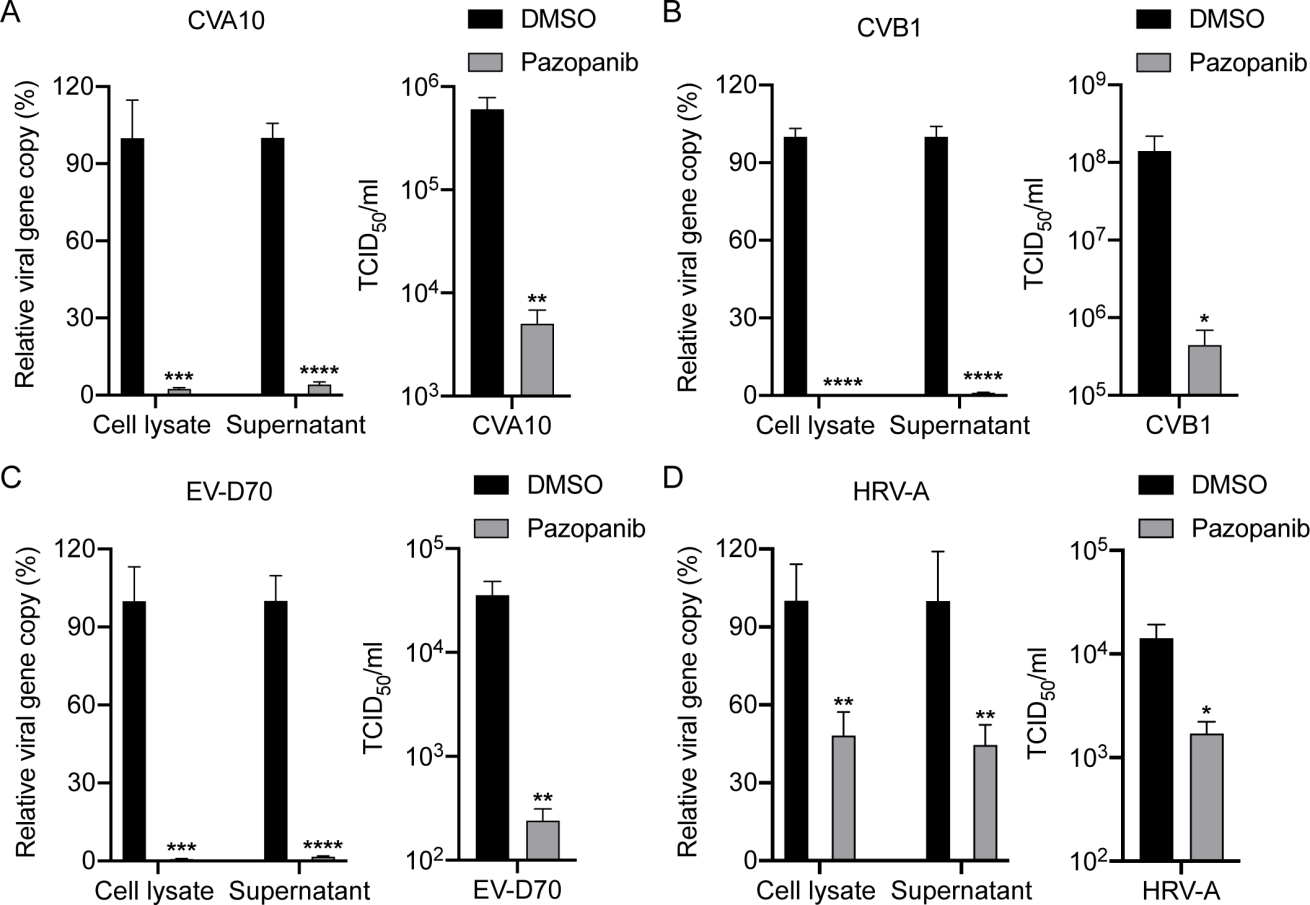
Pazopanib can suppress the replication of four subtypes of enteroviruses. (**A**) RD cells treated with Pazopanib (10 µM) were infected with CVA10. (**B**) BGMK cells treated with Pazopanib (10  µM) were infected with CVB1. (**C**) RD cells treated with Pazopanib (10 µM) were infected with EV-D70. (**D**) MRC-5 cells treated with Pazopanib (10  µM) were infected with HRV-A. After infection with 0.1 MOI of each virus for 24 h, cell lysates and supernatants were harvested for the quantification of viral gene copy number using RT-qPCR analysis. The viral gene copy in the cell lysate is normalized with the transcript of GAPDH and presented. Viral titers were also detected by TCID_50_ assay. Data show the mean and SD of three independent experiments. *P < 0.05; **P < 0.01; ***P < 0.001. ****P < 0.0001. All experiments were independently repeated thrice.

To assess the potential broad-spectrum inhibitory activity of Pazopanib against other enteroviruses, we selected CVA10, CVB1, and EV-D70 from species Enteroviruses A, B, and D, respectively, as well as HRV-A, for the determination of antiviral activity. As expected, consistent with EV-A71 from the Enterovirus A species described above, Pazopanib significantly reduced the viral load and viral titer of CVA10 in cell lysates and supernatants, respectively (Fig. 4A). Of note, Pazopanib exhibited potent antiviral activity against CVB1 and EV-D70, resulting in over 2 logs decrease in viral load and viral titer (Fig. 4B and C). More importantly, a slight but significant inhibition of HRV-A replication was observed upon Pazopanib addition, as evidenced by markedly reduced viral loads in both cell lysate and supernatant (Fig. 4D). The different antiviral effects of Pazopanib may be due to the genetic distance and differences in virological characteristics between human enteroviruses and rhinoviruses. Taken together, in addition to EV-A71, Pazopanib also demonstrated cross-protection against CVA10, CVB1, EV-D70, and HRV-A, suggesting the potential for safe usage of Pazopanib in therapeutic settings as a broad-spectrum antiviral agent against pan-enteroviruses.

### Influence of different treatment conditions of Pazopanib on EV-A71 infection

**Fig 5:**
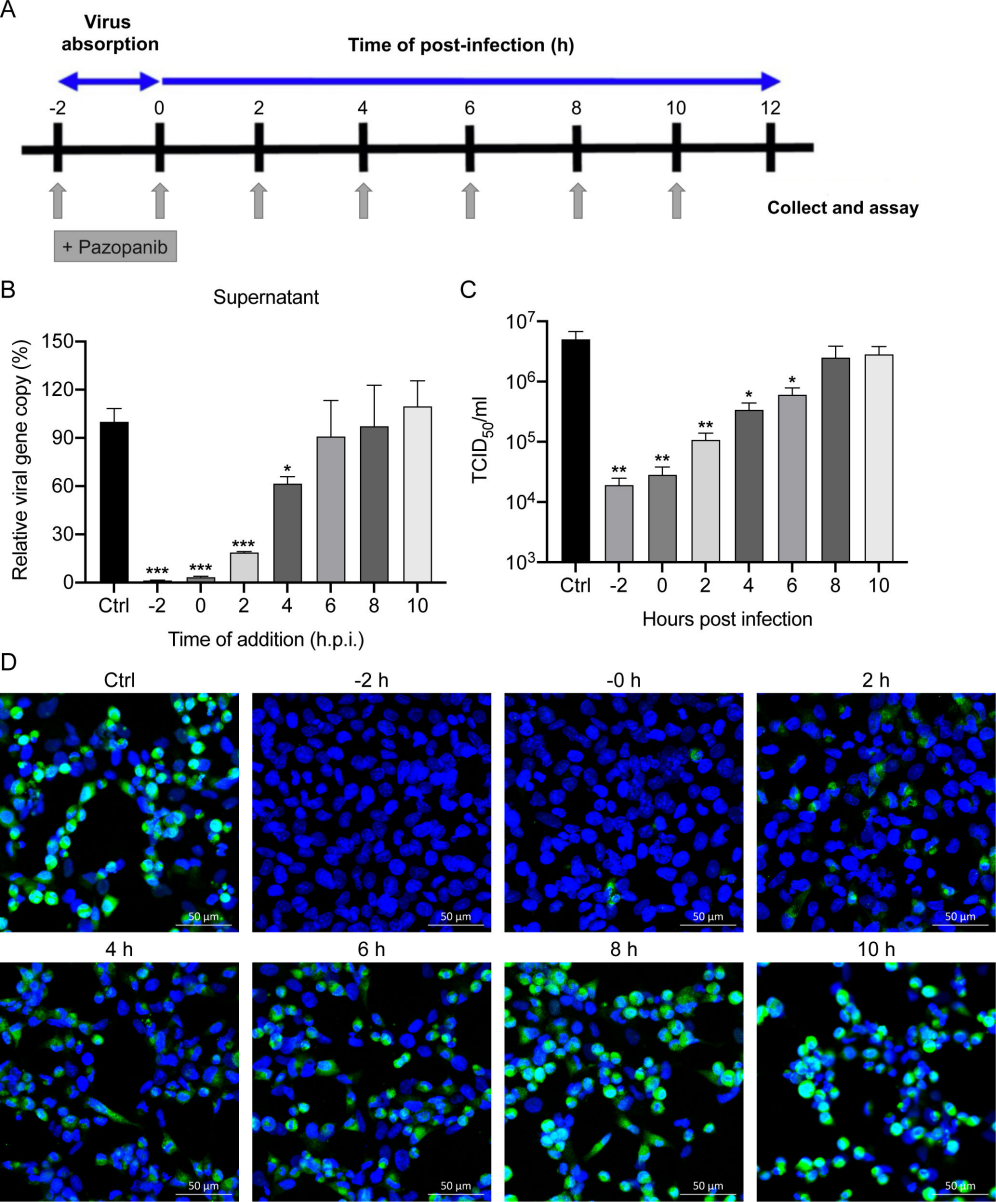
Time-of-drug addition of pazopanib on EV-A71 infection. RD cells were infected with EV-A71 at an MOI of 1, and treated with Pazopanib (10 µM) at indicated time points, including pre-infection (−2 h), co-infection (0 h), and post-infection (2 h, 4 h, 6 h, 8 h, or10h). (**A**) Graphical scheme illustrating the experimental design. (**B**) RT-qPCR measured the amount of viral RNA released into culture supernatant. (**C**) Viral titer was determined in TCID_50_ assays. Data show the mean and SD of three independent experiments. *P < 0.05; **P < 0.01; ***P < 0.001. (**D**) Representative images of VP1 expression by indirect immunofluorescence assay for Pazopanib, using Rabbit anti‐VP1 antibody (green). Nuclei are counterstained with DAPI (blue). Scale bar, 50 µm.

To investigate the stage(s) at which Pazopanib exerted its inhibition actions *in vitro*, the time-of-drug-addition assay was performed as described previously (34). RD cells were treated with Pazopanib at various time points before, concurrently with, or after EV-A71 virus infection, as depicted in Fig. 5A. Considering that the replication cycle of EV-A71 typically occurs within 6–8 h, we collected samples and determined the anti-EV71 activity at 12 hpi. As shown in Fig. 5B and C, the viral load and viral titer of progeny virions in the culture medium were significantly reduced in RD cells at earlier Pazopanib treatment time points (−2 h, 0 h, 2 h, and 4 h), with the reduction being more remarkable with earlier treatment. In contrast, Pazopanib showed no significant inhibitory effect when the infected cells were treated at 6 to 10 hpi. Additionally, the expression of the EV-A71 structural protein VP1 was also affected upon Pazopanib addition, as observed by indirect immunofluorescence assay (Fig. 5D). Thus, these results indicated that Pazopanib primarily inhibits EV-A71 replication effectively during the early stages of viral infection.

To further dissect the anti-EV-A71 mechanism of Pazopanib, we performed viral inactivation, and viral attachment/entry assay to investigate whether the drug directly inactivated the viral particles by binding or inhibiting viral attachment/entry to the host cell surface. As shown in Fig. S5A, the EV-A71 virus titers did not significantly differ between the Pazopanib-treated and DMSO-treated groups, suggesting that Pazopanib had no virucidal effect. Moreover, we found that the EV-A71 mRNA levels in cell lysates did not significantly differ between the Pazopanib-treated and DMSO-treated groups, thus indicating that Pazopanib did not affect the viral attachment/entry stage (Fig. S5B and C). Altogether, these findings suggest that Pazopanib probably induces alterations in host cells, thereby impeding viral genome replication and transcription, rather than inhibiting entry through receptor binding or targeting viral functional proteins, such as protease or polymerase.

### VEGFR2 is essential for EV-A71 replication

**Fig 6:**
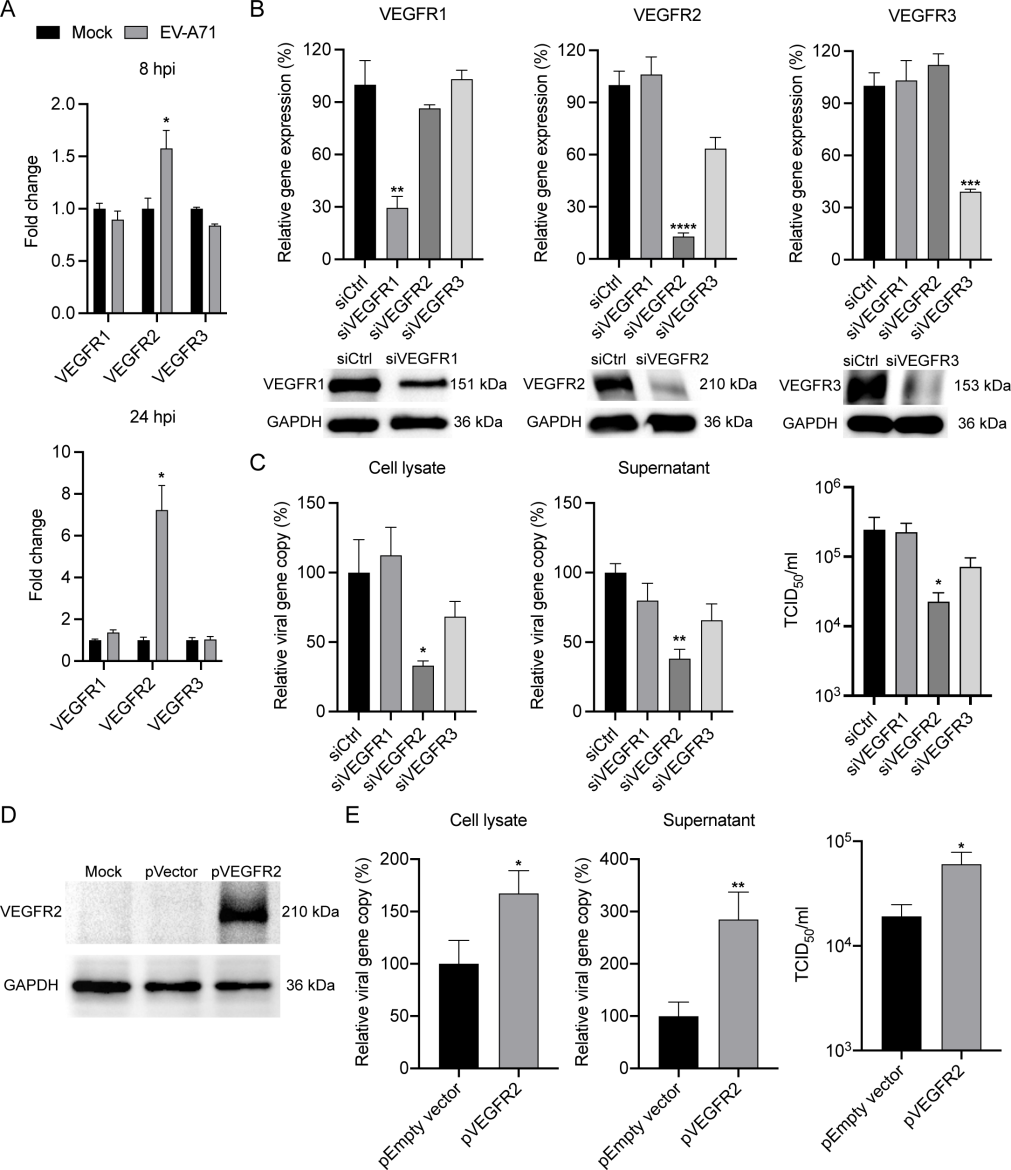
VEGFR2 is inducible upon EV-A71 infection and required for EV-A71 replication. (**A**) At 8 and 24 hpi, the EV-A71-infected RD cells and mock-infected cells were harvested for detection of mRNA expression levels of VEGFRs. Results show the fold change of GAPDH-normalized expression level in the infected cells relative to that in the mock-infected cells. (**B**) siRNA targeting VEGFR1 or VEGFR2 or VEGFR3 or scrambled siRNA (siCtrl) ws transfected into HUVEC cells in 2 consecutive days. At day 3 after siRNA transfection, the cells were harvested to assess the knockdown effect of VEGFR1, VEGFR2, and VEGFR3 by RT-qPCR assay and Western blotting. (**C**) The depleted cells were then infected with EV‐A71 at an MOI of 0.01. At 24 hpi, cell lysate (left) and supernatant (middle) were harvested for the quantification of viral gene copy number using RT‐qPCR analysis. The viral gene copy in the cell lysate is normalized with the transcript of GAPDH and presented. Viral titer (right) was also determined for siRNA‐transfected samples in TCID_50_ assays. (**D**) The plasmids pVEGFR2 or pEmpty vector were transfected into 293T cells. At day 2 post-transfection, the cells were harvested to detect the expression level of VEGFR2 by Western blotting. (**E**) The transfected cells were then infected with EV‐A71 at an MOI of 0.01. At 24 hpi, cell lysate (left) and supernatant (middle) were harvested for the quantification of viral gene copy number using RT‐qPCR analysis. The viral gene copy in the cell lysate is normalized with the transcript of GAPDH and presented. Viral titer (right) was also determined for plasmid‐transfected samples in TCID_50_ assays. Data show the mean and SD of three independent experiments. *P < 0.05; **P < 0.01; ***P < 0.001.

As aforementioned, three isoforms of VEGFR, namely VEGFR1, VEGFR2, and VEGFR3, have been identified, each with specific functions and expression patterns (18). However, the specific gene involved in EV‐A71 replication remains undefined, as the VEGFR inhibitor Pazopanib targets all isoforms indiscriminately. Thus, we firstly measured the mRNA profiles of VEGFRs in EV-A71-infected RD cells. As shown in Fig. 6A, the mRNA expression levels of VEGFR2, but not VEGFR1 and VEGFR3, were significantly upregulated at both 8 and 24 hpi, suggesting an accelerating activation of VEGFR2 during EV-A71 infection.

Next, to delineate the role of VEGFR1, VEGFR2, and VEGFR3 in EV‐A71 replication, we depleted each gene by siRNA knockdown in HUVEC cells and assessed their impact on EV‐A71 replication. The effective depletion of VEGFR1, VEGFR2, and VEGFR3 was confirmed by RT‐qPCR assay at 48 h post siRNA transfection, with the mRNA expression levels of each gene reduced to 20%–30% relative to those in the control cells (Fig. 6B). Meanwhile, these reductions were further verified by Western blot analysis. Subsequently, we infected the transfected cells with EV‐A71, and harvested the cell lysates and supernatants at 24 hpi. As shown in Fig. 6C, a significant reduction in viral load and viral titer was observed in the VEGFR2-depleted cells, whereas no significant reduction was observed in the cells depleted of VEGFR1 and VEGFR3, as evidenced by markedly decreased viral load and viral titer in both cell lysate and supernatant. To further confirm whether VEGFR2 is involved in the replication of EV-A71, we overexpressed the VEGFR2 protein by transfecting a VEGFR2-expressing plasmid into 293T cells. After transfection, Western blot analysis was utilized to detect VEGFR2 protein expression levels, and the transfected cells were utilized for subsequent infection experiments (Fig. 6D). Strikingly, a significant increase in viral load and viral titer was observed in the cells with VEGFR2 overexpression compared with the control group (Fig. 6E). Therefore, these findings suggest that VEGFR2 may be an essential host protease for EV‐A71 replication.

### VEGFR2 may exert its antiviral effects through the TSAd-Src-PI3K-Akt pathway

**Fig 7:**
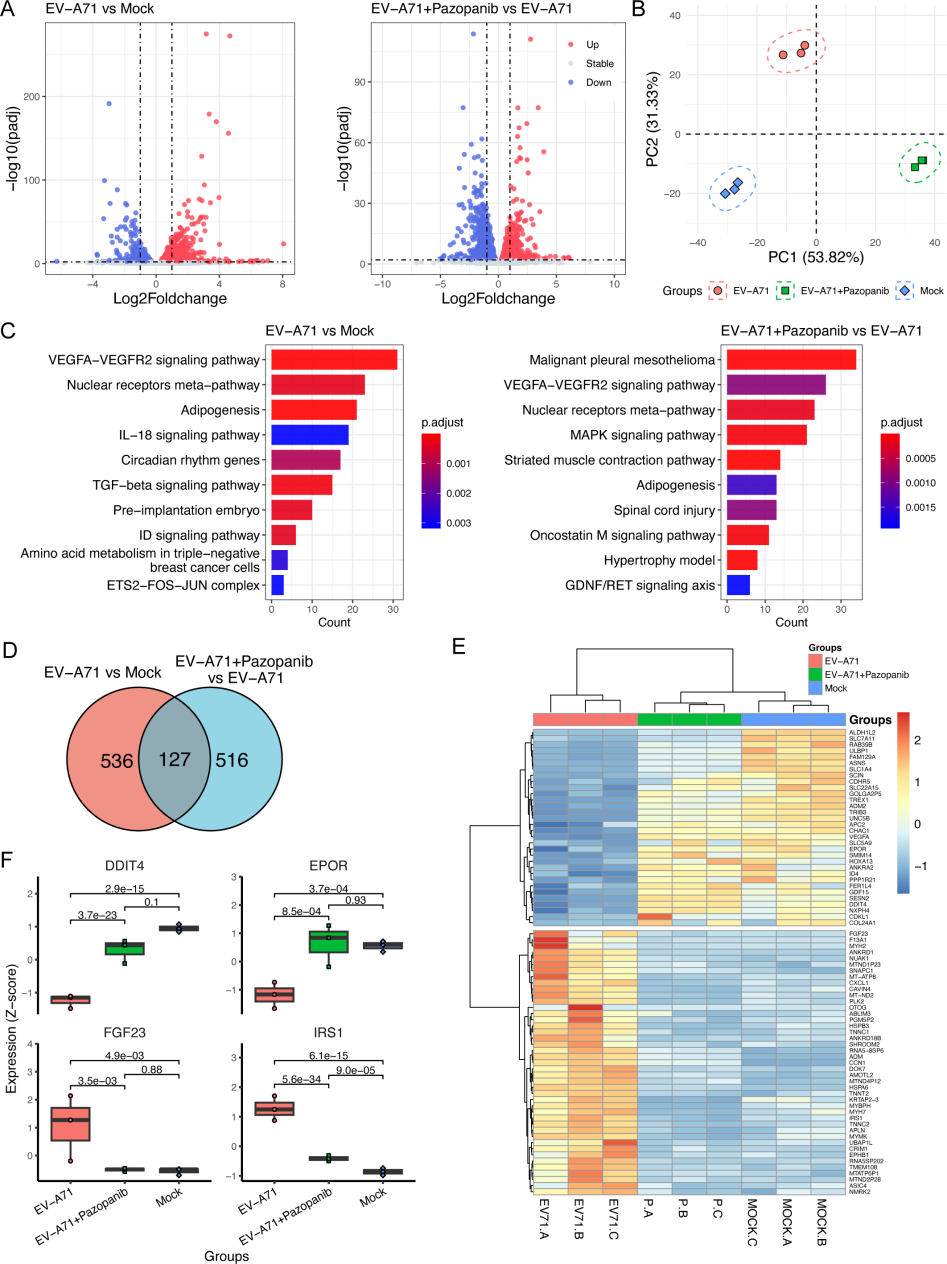
Transcriptome analysis identifies the differential expressed genes in EV-A71-infected cells. (**A**) The EV-A71 (MOI = 0.1) infected or non-infected RD cells were treated with or without Pazopanib (10 µM) for 24 h. Then, the purified RNAs isolated from three experimental groups (mock, EV-A71, EV-A71 + Pazopanib) were subjected to transcriptome analysis. Volcano plots indicate upregulated (red) and downregulated (blue) mRNA transcripts in the EV-A71 vs mock group or the EV-A71 + Pazopanib vs EV-A71 group. (**B**) PCA plot of DEGs showed distinct differences among three groups and high consistency within each group. (**C**) The significantly enriched pathways of DEGs in EV-A71 vs mock (left) and EV-A71 + Pazopanib vs EV-A71 (right). (**D**) The KEGG annotation analysis was performed to display the 86 DEG-related biological processes in EV-A71-infected cells (mock vs EV-A71). Venn analysis indicates about 86 DEGs, which exhibited similar changes in the Mock and EV-A71 + Pazopanib groups, compared with EV-A71 groups. (**E**) The heatmap of gene expression of the 86 DEGs among three groups. (**F**) The boxplots showed different expressions of genes in PI3K-Akt signaling pathways.

To further investigate the host response to virus infection, we analyzed the transcriptomic profile of RD cells infected with EV-A71 in the presence or absence of Pazopanib. As shown in Fig. 7A, about 418 upregulated genes and 245 downregulated genes were identified in the EV-A71-infected group (EV-A71) relative to the Mock-infected group (Mock), with a fold change of ≥1 and *P* < 0.01. However, after Pazopanib treatment, approximately 285 upregulated genes and 358 downregulated genes were identified in the Pazopanib-treated infection group (EV-A71 +Pazopanib) relative to the EV-A71-infected group. Furthermore, the principal component analysis (PCA) plot of differential expression proteins (DEPs) demonstrated distinct differences among the three groups and high consistency within each group, as illustrated in Fig. 7B. Of note, the fold changes of genes in EV-A71 + Pazopanib versus EV-A71 exhibited a significant negative correlation with those in EV-A71 versus mock, suggesting that Pazopanib has the potential to reverse the differential gene expression induced by viral infection (Fig. S6). Additionally, KEGG annotation analysis was performed to further display the differentially expressed gene (DEG)-related biological processes (Fig. 7C). Apparently, the VEGFRA–VEGFR2 signaling pathway appeared to be significantly enriched, whether with EV-A71 infection alone or in the presence of Pazopanib. Intriguingly, there were 75 DEGs exhibiting reverse regulations among these three groups, in which 43 upregulated genes in the EV-A71 versus mock group were downregulated in the EV-A71 + Pazopanib versus EV-A71 group, suggesting their potential involvement in Pazopanib’s inhibitory effect on EV-A71 infection in RD cells (Fig. 7D and E). Among them, we identified four DEGs that were either downregulated or upregulated after EV-A71 infection and returned to normal levels after Pazopanib treatment in RD cells (Fig. 7F). Notably, these genes are all related to the PI3K-Akt signaling pathway, suggesting that Pazopanib may exert its antiviral effects via downstream modulation of the PI3K-Akt signaling pathway.

As aforementioned, VEGFR2 is primarily expressed in vascular endothelial cells and acts as a key signal transducer of angiogenesis via the TSAd-Src-PI3K-Akt pathways. Therefore, we suspect that VEGFR2 exerts antiviral effects via this signaling pathway. To this end, we assessed EV-A71 replication in the presence or absence of Dasatinib, a highly potent Src inhibitor. As shown in Fig. S7A and B, the viral load and viral titer of EV‐A71 were significantly reduced by noncytotoxic concentration of Dasatinib in both cell lysates and supernatant, respectively. Furthermore, Dasatinib markedly decreased EV‐A71 structural protein VP1 expression, as observed in the Western blot analysis (Fig. S7C). Moreover, MK-2206, an Akt1/2/3 inhibitor, also significantly suppressed EV-A71 replication, as evidenced by markedly reduced viral loads in both cell lysate and supernatant (Fig. S8). Meanwhile, as expected, MK-2206 significantly inhibited Akt phosphorylation without altering total Akt levels following EV-A71 infection. Next, to assess whether Pazopanib treatment impairs the activation of the PI3K-Akt pathway, we examined the expression of Akt and phosphorylated Akt in mock-infected or EV-A71-infected cells treated with Pazopanib or not. As shown in Fig. S9, pretreatment with Pazopanib significantly attenuated Akt phosphorylation in mock-infected cells, as revealed by Western blotting. Notably, EV-A71-stimulated Akt phosphorylation was also inhibited by Pazopanib, which collectively indicate that Pazopanib impairs the activation of the PI3K-Akt pathway in both mock-infected and EV-A71-infected cells. More importantly, significant activation of phosphorylated VEGFR2 was observed in EV-A71-infected HUVEC cells by Western blotting, indicating that the VEGF pathway was activated following EV-A71 infection. Taken together, these findings suggest that VEGFR2 may regulate EV-A71 replication by activating the TSAd-Src-PI3K-Akt signaling pathway.

## DISCUSSION

Since the first clinical case was identified in the United States, EV-A71, an important neurotropic enterovirus, has been a common cause of HFMD disease in infants and young children (35). To date, no approved target is available for the treatment of EV-A71 infections, let alone one with broad-spectrum antiviral capabilities (36). As an obligate intracellular pathogen, EV-A71 depends on various cellular factors to complete its life cycle (37). Host kinases play pivotal roles in regulating multiple signaling pathways in response to various stimuli, including viral infections. Notably, TRIB3 can promote EV-A71 infection through dual mechanisms, and MINK, an IRES-mediated protein, participates in EV-A71 replication (11, 12). These findings suggest that host kinases may facilitate EV-A71 infection and could serve as potential targets for developing antiviral agents against EV-A71. Traditionally, immortalized cell lines are widely used as screening platform in the development of antiviral drugs (38, 39). However, drugs selected using this platform may not be appropriate for *in vivo* studies, as cell lines cannot fully recapitulate the cellular diversity and complex functions of human physiology and disease pathology (40). Human enteroviruses are primarily transmitted *via* the fecal–oral route, with the intestinal epithelium being the primary target of viral invasion. Additionally, it remains unclear how EV-A71 occasionally involves the central nervous system and induces diverse neurological complications. To this end, we previously conducted simultaneous screening of a protein kinase inhibitor library using RD cells and 2D human intestinal organoids to identify cellular kinases required for EV-A71 viral growth. We found that GSK269962A, a Rock inhibitor, exhibits antiviral effects, and depletion of Rock1 significantly reduces EV-A71 replication, indicating that Rock1 is a host-dependent factor involved in EV-A71 replication.

In this study, we further found that various VEGFR inhibitors, including Pazopanib, Brivanib, SU14813 and Axitinib, exhibit potent antiviral effects in both RD cells and 2D human intestinal organoids, as evidenced by CPE inhibition and viral reduction assays (Fig. 1). As aforementioned, the VEGFR family has long been an important target for tumor therapy. Pazopanib, Brivanib, and Axitinib have been approved for clinical use to treat cancers, suggesting the potential of repurposing these drugs as therapeutics against EV-A71 infection (41). Among these inhibitors, Pazopanib demonstrated the highest inhibitory activity against EV-A71 infection, with a selectivity index exceeding that of Pirodavir, a potent broad-spectrum picornavirus inhibitor targeting viral capsid protein VP1 (Table 1). Additionally, Pazopanib not only efficiently suppressed EV-A71 replication in a dose-dependent manner but also provided cross-protection against CVA10, CVB1, EV-D70, and HRV-A, suggesting its potential as a broad-spectrum antiviral agent for pan-enteroviruses (Fig. 2 and 4). In contrast, a recent study demonstrated that administration of Pazopanib ameliorates murine hepatitis virus strain 1-induced acute lung injuries without affecting viral replication, suggesting that the antiviral effect of Pazopanib may be virus-specific (42).

Pazopanib has been characterized as a potent VEGFR inhibitor that competes with adenosine triphosphate (ATP) for binding to the intracellular side of tyrosine kinase receptors, preventing ATP-induced activation, with IC_50_ values of 10, 30, and 47 nM for VEGFR1, VEGFR2, and VEGFR3, respectively (43). Although Pazopanib is associated with adverse events, such as liver dysfunction and hypertension, it is now considered an important treatment option for advanced soft-tissue sarcoma and renal cell carcinoma (44). Previous studies have shown that over-expressed VEGFR2 has been detected in melanoma, thyroid cancer, and other solid tumors (45). Despite the three VEGFR isoforms displaying similar primary structures, growing evidence suggests that individual knockout of VEGFR1, VEGFR2, and VEGFR3 results in nonredundant functions *in vitro* and *in vivo* (46). Interestingly, we also found that cellular mRNA of VEGFR2, but not VEGFR1 or VEGFR3, was significantly increased after EV-A71 infection in RD cells (Fig. 6). We then investigated the specific effects of the three cytosolic isoforms on viral growth via genetic depletion. Notably, siRNA depletion of VEGFR2, but not VEGFR1 or VEGFR3, significantly reduced EV-A71 replication in both cell lysates and supernatant. Furthermore, overexpression of VEGFR2 promoted EV-A71 replication, indicating that VEGFR2 is a potential host factor involved in EV-A71 replication efficiency.

Previous studies have shown that VEGFR2 is primarily distributed in vascular endothelial cells and acts as a major signal transducer for angiogenesis through the TSAd-Src-PI3K-Akt, PLCγ-PKC-eNOS-NO, PLCγ-PKC-MAPK, NCK-p38-MAPKAPK2/3, SHB-FAK-paxillin, and SHB-PI3K-Rac pathways (47). Notably, VEGFR2 regulates endothelial cell survival mainly by activating the TSAd-Src-PI3K-PKB/AKT signaling pathway (48). In this study, transcriptome analysis showed that the VEGFRA–VEGFR2 signaling pathway potentially plays a crucial role in combating EV-A71 infection in the presence of Pazopanib or not (Fig. 7). Furthermore, four DEGs that were either downregulated or upregulated after EV-A71 infection returned to normal levels after Pazopanib treatment in RD cells, all related to the PI3K-Akt signaling pathway. This suggests that Pazopanib may exert its antiviral effects *via* downstream modulation of the PI3K-Akt pathway. Moreover, the Src inhibitor Dasatinib and the Akt inhibitor MK-2206 both exerted anti-EV-A71 replication effects in RD cells, as evidenced by significantly reduced viral loads in both cell lysates and supernatants (Fig. S7 and S8). Meanwhile, Pazopanib impaired the activation of the PI3K-Akt pathway in both mock-infected and EV-A71-infected cells (Fig. S9). More importantly, significant activation of phosphorylated VEGFR2 was observed in EV-A71-infected HUVEC cells by Western blotting, indicating that the VEGF pathway was activated following EV-A71 infection. Taken together, these results suggest that the antiviral effect of Pazopanib in EV-A71 infection is most likely due to the TSAd-Src-PI3K-Akt signaling pathway mediated by VEGFR2.

However, our experiments have some limitations. First, although we demonstrated that Pazopanib has broad-spectrum anti-enterovirus effects, further elucidation is needed to determine if its structural analogs have similar antiviral effects to exclude side effects. Second, targeting the host factor VEGFR2 may result in toxic reactions. Nevertheless, toxicity could be mitigated by modifying the drug’s structure. Third, the broad-spectrum anti-enteroviral effects of Pazopanib have been confirmed *in vitro* but not *in vivo*, warranting further studies. Finally, although a total of nine VEGFR inhibitors have been approved by FDA for clinical use, suggesting potential therapeutic applicability in a wide variety of pathological conditions, the safety of Pazopanib remains unclear. Future studies should evaluate the safety profile of Pazopanib through side-by-side comparisons in both *in vitro* and *in vivo* studies.

In summary, we demonstrated that VEGFR inhibitor Pazopanib not only efficiently suppressed the replication of EV-A71 in a dose-dependent manner, but also exhibited broad-spectrum anti-enterovirus activity. More importantly, VEGFR2 knockdown and overexpression suppressed and facilitated EV-A71 replication, respectively, indicating that VEGFR2 is a novel host dependency factor for EV-A71 replication. In addition, transcriptome analysis further proved that VEGFR2 potentially plays a crucial role in combating EV-A71 infection through the TSAd-Src-PI3K-Akt pathway. The knowledge obtained from this study will provide a scientific basis for understanding the function of VEGFR2 in combating EV-A71 infection, aiding the development of prophylactics and therapeutics against enterovirus infections.

## Data Availability

All data are available upon request, and inquiries should be sent to xiaoyu_zhao@fudan.edu.cn.

## SUPPLEMENTAL MATERIAL

Supplemental figures and tables jvi.01129-24-s0001.docx Figures S1 to S9; Table S1.

ASM does not own the copyrights to Supplemental Material that may be linked to, or accessed through, an article. The authors have granted ASM a non-exclusive, world-wide license to publish the Supplemental Material files. Please contact the corresponding author directly for reuse.
