# Supplementary material for: Vascular endothelial growth factor receptor 2 as a potential host target for the inhibition of enterovirus replication
Source: J Virol. 2024 Sep 17;98(10):e01129-24. doi: 10.1128/jvi.01129-24 (PMC11542593; doi:10.1128/jvi.01129-24)
Supplement: Supplemental figures and tables — Figures S1 to S9; Table S1. [file jvi.01129-24-s0001.docx]

**Supplementary Materials**

**Fig. S1.** (**A**) RD cells were treated with the indicated concentrations of inhibitors and incubated at 37°C for 24 h. CellTiter‐Glo® luminescent cell viability assay was used to measure the CC_50_ of selected inhibitors. Data show the mean and SD of three independent experiments. (**B**) RD cells treated with the selected inhibitors (10 μM) were inoculated with EV-A71 at an MOI of 0.01. At 48 hpi, cells were harvested to detect the expression level of VP1 by western blotting.

**Fig. S2.** RD cells treated with the indicated concentration of VEGFR inhibitors, including Axitinib (**A**), Brivanib (**B**) and SU14813 (**C**), were inoculated with EV‐A71 at an MOI of 0.1 for 24 h. Dose-dependent inhibition of EV-A71 infection was found for both cell lysate (left) and supernatant (right) using RT-qPCR analysis. The results represent the viral load in the cells treated with indicated concentration of VEGFR inhibitors relative to that in DMSO-treated cells. Viral titer was also determined for samples treated with indicated concentrations of VEGFR inhibitors in TCID_50_ assays. Data show the mean and SD of three independent experiments. ns, Not Statistically Significant; *p < 0.05; **p < 0.01; ***p < 0.001. ****p < 0.0001.

**Fig. S3.** RD cells treated with the indicated concentration of VEGFR inhibitors, including Axitinib (**A**), Brivanib (**B**) and SU14813 (**C**), were inoculated with EV‐A71 at an MOI of 0.1 for 24 h. VP1 protein levels were measured for samples treated with indicated concentrations of VEGFR inhibitors using western blotting.

**Fig. S4.** The EV-A71 (MOI =2) infected or non-infected RD cells were treated with or without Pazopanib (10 μM). Then the infected- and mock-infected RD cells were fixed, labeled, and applied to flow cytometry to evaluate the percentage of VP1-positive cells at 0, 3, 6 and 9 hpi. The histogram shows the results of one representative experiment (**A**). Mean and SD of three independent experiments are presented (**B** and **C**). *p < 0.05; **p < 0.01.

**Fig. S5.** **Pazopanib not interferes with EV-A71 entry into cells.** (**A**) EV-A71 inactivation assay. EV-A71 was incubated with Pazopanib (10 µM) for 2 h at 25°C, followed by TCID_50_ assays. (**B**) EV-A71 attachment assay. RD cells were pre-treated with Pazopanib for 4 h at 37°C, followed by thorough washing and transfer to a 4°C environment for incubation with EV-A71 (MOI = 5). After 2 hpi, the infectious inoculum was removed and cells were washed again. The intracellular viral RNA load was then determined using RT-qPCR analysis. (**C**) EV-A71 entry assay. RD cells were infected with the mixture of EV-A71 (MOI = 5) and Pazopanib for 2 h, followed by thorough washing and detection of intracellular EV-A71viral gene copy by RT-qPCR analysis. Data show the mean and SD of three independent experiments. ns, Not Statistically Significant.

**Fig. S6.** The fold changes of genes in EV-A71+Pazopanib vs EV-A71 group showed a significant negative correlation with those in EV-A71 vs Mock group.

**Fig. S7. Viral reduction in RD cells with Src inhibitor treatment.** (**A**) RD cells were treated with the indicated concentrations of Dasatinib and incubated at 37°C for 24 h. CellTiter‐Glo® luminescent cell viability assay was used to measure the CC_50_ of Dasatinib. (**B**) RD cells treated with the Dasatinib (10 μM) were inoculated with EV-A71 at an MOI of 0.01 for 24 h. Significant inhibition of EV-A71 infection was found for both cell lysate (left) and supernatant (middle) using RT-qPCR analysis. The results represent the viral load in the cells treated with Dasatinib relative to that in DMSO-treated cells. Viral titer was also determined for inhibitor-treated samples in TCID_50_ assays (right). Data show the mean and SD of three independent experiments. *p < 0.05; **p < 0.01. (**C**) VP1 protein levels were measured for samples treated with DMSO or Dasatinib using western blotting.

**Fig. S8. Viral reduction in RD cells with Akt inhibitor treatment.** (**A**) RD cells were treated with the indicated concentrations of MK-2206 and incubated at 37°C for 24 h. CellTiter‐Glo® luminescent cell viability assay was used to measure the CC_50_ of MK-2206. (**B**) RD cells treated with the MK-2206 (10 μM) were inoculated with EV-A71 at an MOI of 0.01 for 24 h. Significant inhibition of EV-A71 infection was found for both cell lysate (left) and supernatant (middle) using RT-qPCR analysis. The results represent the viral load in the cells treated with MK-2206 relative to that in DMSO-treated cells. Viral titer was also determined for inhibitor-treated samples in TCID_50_ assays (right). Data show the mean and SD of three independent experiments. *p < 0.05; **p < 0.01; ***p < 0.001. (**C**) The levels of VP1, phosphorylated Akt and total Akt protein were measured for samples treated with DMSO or MK-2206 using western blotting.

**Fig. S9. The expression of Akt and Akt phosphorylation in Mock-infected and EV-A71-infected cells treated with Pazopanib or not.** (**A**) RD cells were infected with EV-A71 at an MOI of 1, and cell lysates were collected at the indicated times. Western blotting assays were then performed to assess the levels of phosphorylated Akt and total Akt. (**B**)The plasmids pVEGFR2 or pEmpty vector were transfected into RD cells. At the indicated times post-transfection, cells were harvested to detect the expression levels of phosphorylated Akt and total Akt by western blotting. (**C**) RD cells were treated with Pazopanib (10 µM) or not, and cell lysates were collected at the indicated times. Western blotting assays were then performed to assess the levels of phosphorylated Akt and total Akt. (**D**) RD cells treated with Pazopanib (10 µM) were infected with EV-A71 at an MOI of 1. At 1 hpi, cells were harvested to detect the expression levels of phosphorylated Akt and total Akt by western blotting. (**E**) The plasmids pAkt or pEmpty vector were transfected into 293T cells. At day 1 post-transfection, the transfected cells were then infected with EV‐A71 at an MOI of 5 in the presence or absence of Pazopanib (10 µM). At 6 hpi, cells were harvested to detect the expression level of VP1, phosphorylated Akt and total Akt by western blotting. (**F**) HUVEC cells were infected with EV-A71 at an MOI of 0.01, and cell lysates were collected at the 24 hpi. Western blotting assays were then performed to assess the levels of phosphorylated VEGFR2 and total VEGFR2.

**Table S1. The primer sequences for RT-qPCR assay.**

| **Gene** | **Type** | **Sequence** |
| --- | --- | --- |
| GAPDH | Forward primer | ATTCCACCCATGGCAAATTC |
|  | Reverse primer | CGCTCCTGGAAGATGGTGAT |
| Pan-EV VP1 | Forward primer | GCCCCTGAATGCGGCTAAT |
|  | Reverse primer | ATTGTCACCATAAGCAGYCA |
| IL-6 | Forward primer | GGTACATCCTCGACGGCATCT |
|  | Reverse primer | GTGCCTCTTTGCTGCTTTCAC |
| IL-8 | Forward primer | GGCACAAACTTTCAGAGACAG |
|  | Reverse primer | ACACAGAGCTGCAGAAATCAGG |
| TNF-α | Forward primer | GGCTCCAGGCGGTGCTTGTTC |
|  | Reverse primer | AGACGGCGATGCGGCTGATG |
| VEGFR1 | Forward primer | TTTGCCTGAAATGGTGAGTAAGG |
|  | Reverse primer | TGGTTTGCTTGAGCTGTGTTC |
| VEGFR2 | Forward primer | GGCCCAATAATCAGAGTGGCA |
|  | Reverse primer | CCAGTGTCATTTCCGATCACTTT |
| VEGFR3 | Forward primer | TGCACGAGGTACATGCCAAC |
|  | Reverse primer | GCTGCTCAAAGTCTCTCACGAA |
